# 1,25-Dihydroxyvitamin D Testing in Clinical Practice: A Literature Review on Diagnostic Challenges Arising From Analytical Variability

**DOI:** 10.7759/cureus.92683

**Published:** 2025-09-19

**Authors:** Amrou Farag, Dushyant Sharma

**Affiliations:** 1 Diabetes and Endocrinology, Royal Liverpool University Hospital, Liverpool, GBR; 2 Diabetes and Endocrinology, Liverpool University Hospitals NHS Foundation Trust, Liverpool, GBR

**Keywords:** 1'25-dihydroxyvitamin d, assay performance evaluation, clinical laboratory assays, immunoassay variability, vitamin d metabolism

## Abstract

As our understanding of vitamin D and its metabolites has improved, different assays have been developed for measuring their serum concentrations. The predominant circulating metabolite, 25-hydroxyvitamin D (25(OH)D), has been extensively studied and is now widely used for assessing vitamin D status. Of current interest is the biologically active metabolite, 1,25-dihydroxyvitamin D (1,25(OH)_2_D), which is involved in numerous physiological processes related to bone metabolism and various non-calcaemic functions. Quantification of 1,25(OH)_2_D serves not only as a diagnostic tool for numerous diseases, but also as a way to ensure optimal levels in the circulation to maintain physiological functioning. Measuring 1,25(OH)_2_D, however, proves challenging as its concentration in the blood is very low, and the molecule itself is highly lipophilic. A variety of analytical assays have been developed for measuring 1,25(OH)_2_D, each differing in its analytical principles. These methods exhibit variations in their performances, showcasing both strengths and limitations in their underlying analytical principles; some of these assays continue to be used today, while others have become obsolete. Comparability of these methods to this day is challenging due to the absence of a standardisation scheme for 1,25(OH)_2_D.

## Introduction and background

Vitamin D, a lipid-soluble 27-carbon secosteroid, is an essential component in calcium and phosphate homeostasis, thus optimising the functioning of the human skeleton [1]. Due to the distribution of the vitamin D receptor (VDR) across many body tissues [2], vitamin D is also involved in numerous non-calcaemic functions [3]. Research on vitamin D has become an area of great demand as our understanding of its effects on physiological and pathological processes has improved. This increasing interest has paved the way for the development of a variety of laboratory techniques to quantify the serum concentration of vitamin D; these range from labour-intensive analytical methods to precise, accurate, and specific automated assays. Assessing serum concentration of vitamin D continues to be a highly controversial matter [4]. One of the primary obstacles pertains to identifying the most clinically relevant metabolites and defining the concentration that constitutes deficiency. Many research efforts have centred on 25-hydroxyvitamin D (25(OH)D), which is the predominant circulating metabolite and accepted marker of vitamin D status [4]. 1,25-dihydroxyvitamin D (1,25(OH)_2_D), by contrast, has comparatively received less attention despite its recognised clinical value and analytical difficulties in measuring it reliably [4]. With the emergence of numerous assays that differ in their analytical principles, uncertainties persist regarding the performance and comparability of these methods for measuring 1,25(OH)_2_D [4].

Physiology of vitamin D

**Figure 1:**
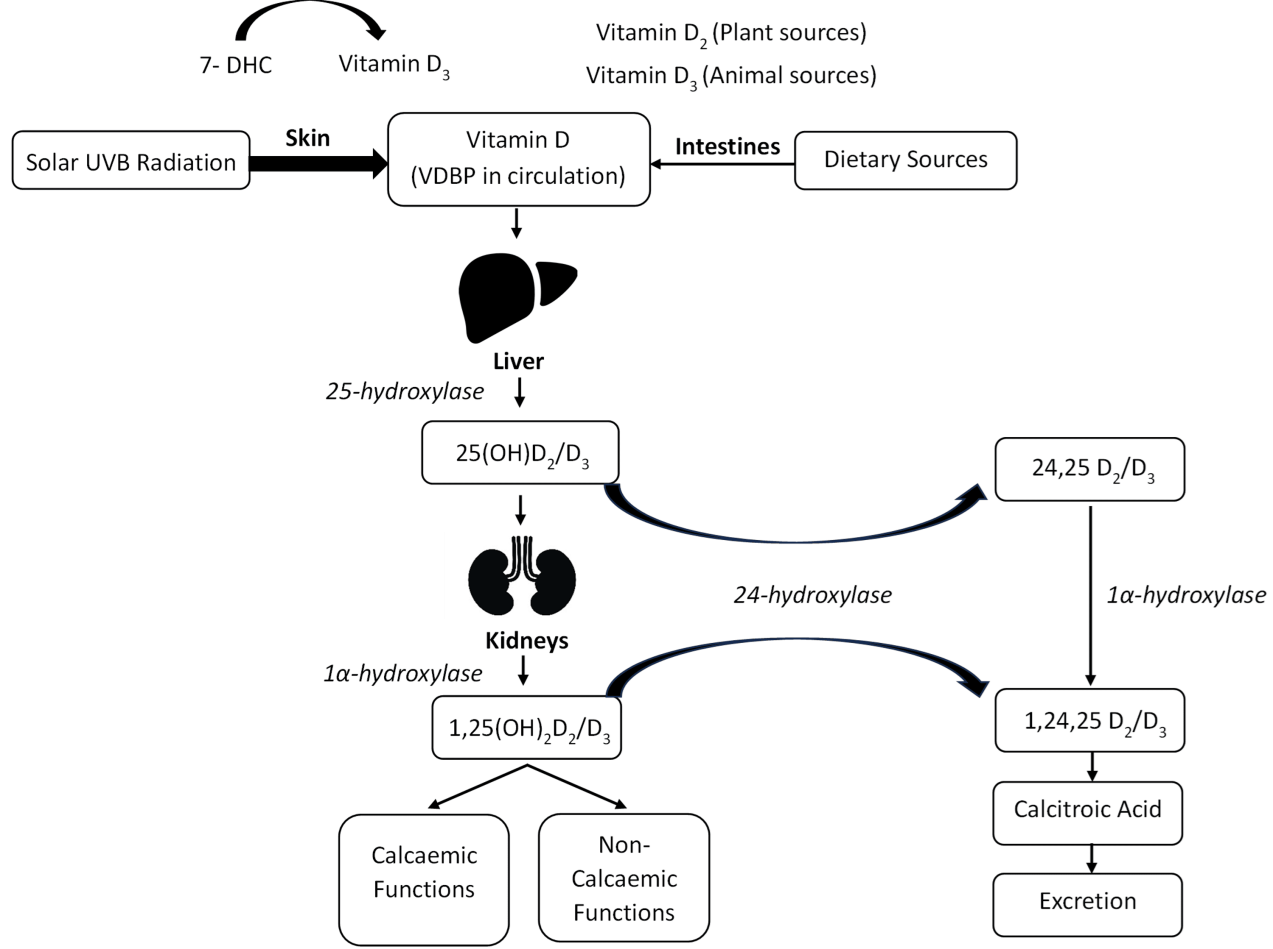
Physiological vitamin D pathway in the body. Figure created by the author A. Farag, based on biological knowledge, and not adapted from any previously published copyrighted source. 7-DHC: 7-dehydrocholesterol; UVB: ultraviolet B; VDBP: vitamin D binding protein

**Figure 2:**
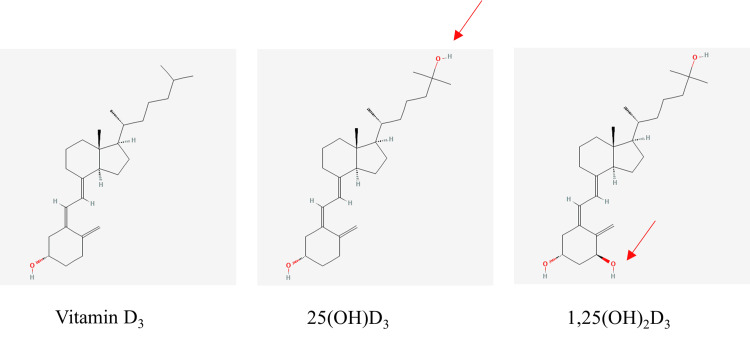
Hydroxylation processes in the liver (middle) and kidneys (right) (red arrows represent the added OH groups in each step). Molecular structures were sourced from PubChem (CIDs: 5280795, 5283731, and 5280453), National Library of Medicine (public domain). Figure annotated by the author (A. Farag) to illustrate the sequential hydroxylation of vitamin D_3_.

Vitamin D exists in two main forms: vitamin D_2_, obtained from plant-based dietary sources, and vitamin D_3_, produced in the skin via sunlight exposure and obtained from animal-based foods (Figure 1). In systemic circulation, vitamin D attaches to the vitamin D binding protein (VDBP) and is transported to the liver, where it undergoes the first hydroxylation by 25-hydroxylase enzyme to form 25(OH)D (Figure 2) [1,4,5]. In the kidneys, 1α-hydroxylase enzyme catalyses another hydroxylation reaction, forming the biologically active form, 1,25(OH)_2_D from 25(OH)D [6] (Figure 2). A proportion of 1,25(OH)_2_D is also broken down by 24-hydroxylase enzyme to form 1,24,25-dihydroxyvitamin D (1,24,25(OH)_2_D) through a negative feedback loop [1,7]. The conversion from 25(OH)D to 1,25(OH)_2_D in the kidneys does not conform to a 1:1 ratio, but a proportion of 25(OH)D gets converted by 24-hydroxylase enzyme (intestinal and renal) to form 24,25-dihydroxyvitamin D (24,25(OH)_2_D) [4,5].

1,25(OH)_2_D is responsible for modulating bone metabolism and mineralisation by interacting with various physiological systems. In the intestines, it facilitates transcellular transport of both calcium and phosphate [8]. In the kidneys, it aids in the reabsorption of calcium and phosphate [9]. 1,25(OH)_2_D also contributes to the fine-tuned control of bone remodelling via the receptor activator of nuclear factor kappa-B ligand (RANK-L) signalling pathway [10]. Furthermore, 1,25(OH)_2_D demonstrates several physiological non-calcaemic functions that encompass modulating immune responses and cellular growth and differentiation [11,12]. Connections between 1,25(OH)_2_D and other systems are also well established in the literature; these include cardiovascular [13], neurological [14], and psychiatric health [15]. This multifaceted impact of 1,25(OH)_2_D highlights its importance not only as a regulator of bone metabolism but also as a key component in broader physiological processes [16].

Vitamin D metabolites and their functions

**Figure 3:**
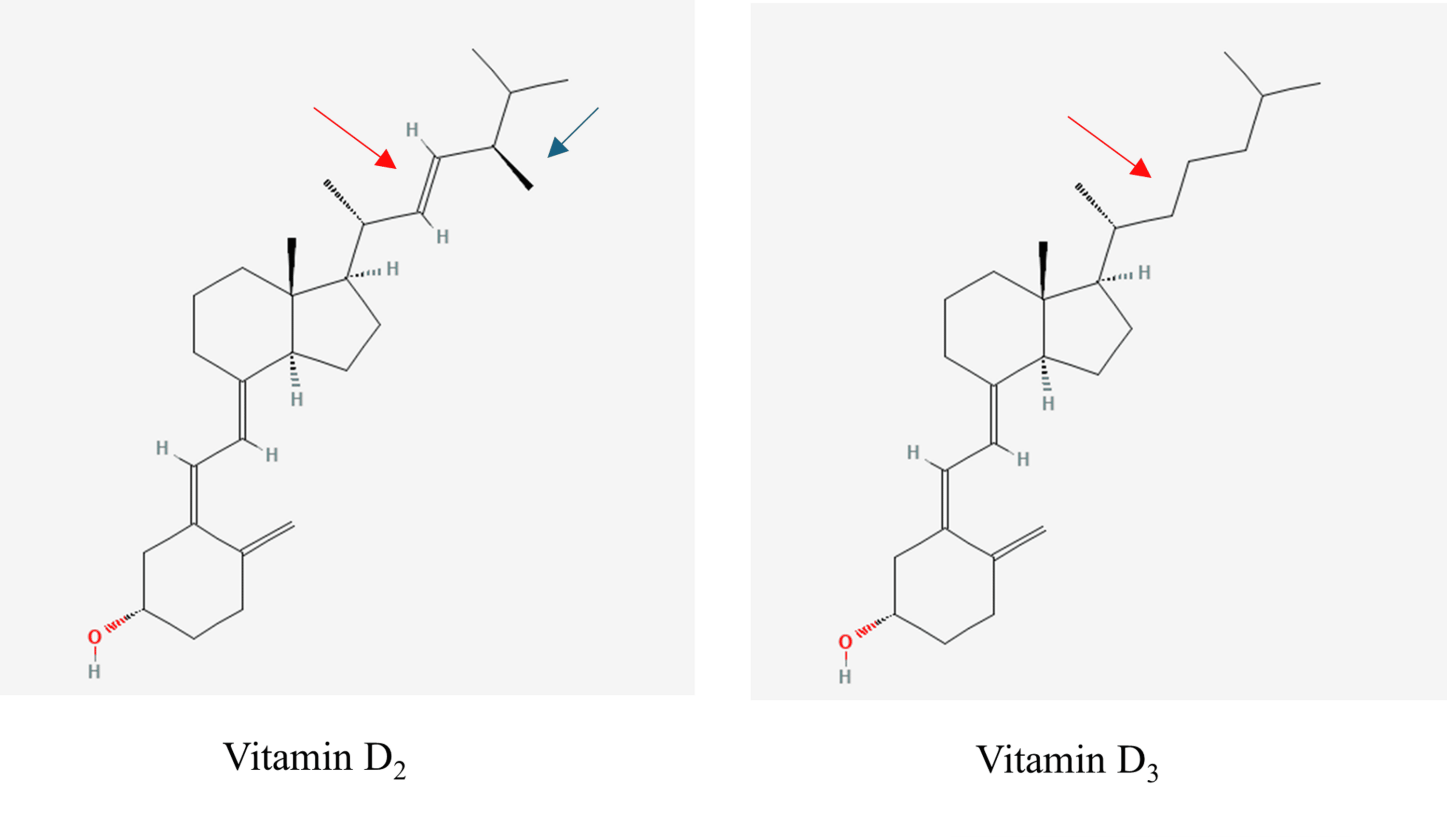
Structure of vitamin D2 and vitamin D3. Vitamin D2 possesses an extra methyl group located at carbon 24 (blue arrow) and a double bond between carbon 22 and 23 (red arrows). Molecular structures were sourced from PubChem (CIDs: 5280731 and 5280795), National Library of Medicine (public domain). Figure annotated by the author (A. Farag).

Vitamin D has a specific cis-triene structure, which makes it susceptible to various chemical reactions, including oxidation and conformational changes [17]. These processes give rise to numerous metabolites exhibiting varying biological activities. Today, vitamin D has more than 40 recognised metabolites [4]. Each of these metabolites exists in both D_2_ (plant-derived) and D_3 _(sunlight and animal-derived forms). Structurally, the D_2_ and D_3_ metabolites of vitamin D differ in their side chains (Figure 3) [18]. Both forms share the same metabolic pathways, but emerging evidence suggests that the D_3_ metabolite may be more efficiently metabolised by the body [19]. The distinction between the D_2_ and D_3_ metabolites is clinically relevant as assays can differ in their comparative ability to detect the two forms.

25(OH)D was first identified in the 1960s and is the primary metabolite used to quantify and assess the serum concentration of vitamin D [20-22]; it has higher circulating concentrations (nanomoles per litre) and longer half-life (21-30 days) compared to the rest of the metabolites [4]. Further research led to the isolation and identification of 1,25(OH)_2_D [23]. The discovery of several other metabolites followed thereafter; however, they have limited clinical application compared to 25(OH)D and 1,25(OH)_2_D [5].

Clinical relevance of 1,25(OH)_2_D

The total serum concentration of 1,25(OH)_2_D (1,25(OH)_2_D_2_ + 1,25(OH)_2_D_3_) is strongly influenced by factors such as calcium, phosphate, fibroblast growth factor-23 (FGF-23), and parathyroid hormone (PTH). Vitamin D deficiency, therefore, may not be necessarily conjoined with a low serum concentration of 1,25(OH)_2_D [1,4], rendering it clinically irrelevant for determining vitamin D status [1,4,24]. 25(OH)D concentration needs to decrease to extremely low levels below 10 nmol/L (4 ng/mL) for 1,25(OH)_2_D concentration to decrease [4]. Instead, quantification of serum 1,25(OH)_2_D is clinically beneficial for informing diagnosis and management of hyperparathyroidism associated with normal 25(OH)D, hypercalcemia precipitated by granulomatous diseases, as well as hereditary mutations like 1 α-hydroxylase deficiency, which causes type 1 vitamin D-dependent rickets (VDDR) and VDR gene mutations, which cause type II VDDR [4,18]. Thus, while 1,25(OH)_2_D lacks validity as a biomarker of vitamin D status, it remains of diagnostic importance in defined pathological conditions. Nevertheless, measuring 1,25(OH)_2_D continues to be constrained by substantial analytical limitations, including its low serum concentration, short half-life, and lack of assay standardisation across laboratories.

Analytical challenges in determining 1,25(OH)_2_D concentration

Intrinsic Measurement Challenges

Measuring 1,25(OH)_2_D concentration poses a more substantial challenge compared to 25(OH)D. The former is highly lipophilic and is present in picomolar concentrations (10^3^-fold lower than the concentration of 25(OH)D) with a far shorter half-life (four to five hours) [4,24]. Several assays have been developed to address these analytical challenges. These include immunoassay-based methods and liquid chromatography with mass spectrometry techniques such as high-performance liquid chromatography (HPLC) and liquid chromatography with tandem mass spectrometry (LC-MS/MS). Different methods have variable specificity and accuracy. For the immunoassay-based methods, their accuracy largely depends on the specificity of the antibody, i.e., its ability to recognise both the D_2_ and D_3_ metabolites [18]. Some LC-MS/MS and HPLC methods, in contrast, can demonstrate better specificity levels towards both metabolites [18].

Standardisation Challenges

Given the availability of numerous analytical methods for measuring vitamin D concentrations, differences were observed amongst measurements obtained from different assays and different laboratories testing the same samples [25]. For 25(OH)D, these observed differences meant the establishment of a standardisation programme to ensure comparability of measurements produced by laboratories using different methods. In 2013, the Vitamin D External Quality Assessment Scheme (DEQAS), in collaboration with the National Institute of Standards and Technology (NIST) and the Vitamin D Standardisation Programme (VDSP), introduced an accuracy-based standardisation scheme for 25(OH)D measurements [26]. As part of this scheme, DEQAS purchases serum samples, and NIST utilises its own LC-MS/MS assay as a reference measurement procedure (RMP) for assigning definitive values of 25(OH)D_2_ and 25(OH)D_3_ to all the samples [26]. These samples are distributed quarterly to many participating laboratories to analyse them and return the measured results. To pass the DEQAS annual proficiency criteria for 25(OH)D, laboratories must ensure that 75% of their results fall within a ±25% range of the total concentration of 25(OH)D (25(OH)D_2_ + 25(OH)D_3_) determined by NIST [27]. Furthermore, the VDSP promotes the usage of standards that can be traced back to the RMPS established by NIST, the Centers for Disease Control and Prevention (CDC), and the University of Ghent to ensure that the measurements obtained across the different laboratories or methods adhere to a certain level of variability [25]. NIST, in collaboration with the CDC, also provides standard reference materials (SRMs) that can contain accurately known concentrations of 25(OH)D_2_ and 25(OH)D_3_ to laboratories for the purpose of calibrating instruments and validating measurement methods [28].

No comparable standardisation programme exists for 1,25(OH)_2_D at the same level achieved for 25(OH)D. As of today, there is no established RMP for 1,25(OH)_2_D, and no SRMs are available for laboratories to calibrate their instruments and validate methods for measuring 1,25(OH)_2_D concentrations [4]. The absence of SRMs has posed challenges in evaluating measurements from various techniques across different laboratories [18]. This lack of rigorous standardisation can undermine clinical confidence in 1,25(OH)₂D testing, particularly in cases where interpretation of results directly informs diagnosis or guides therapeutic decisions. The review included in this paper outlines the available analytical methods used to measure 1,25(OH)_2_D concentration and highlights their strengths and limitations.

## Review

Methodology

Study Design

This narrative review provides an overview of analytical techniques used to quantify serum concentration of 1,25(OH)_2_D, with attention to their limitations and subsequent impact on clinical interpretation and decision making. A literature review was performed to identify studies evaluating analytical methods for measuring 1,25(OH)_2_D. The search was performed using Ovid MEDLINE and Ovid Embase, including articles published in English up to January 2025.

Search terms included both controlled vocabulary (e.g., MeSH: Calcitriol/, Mass Spectrometry/, Immunoassay/) and keywords (e.g., “1,25-dihydroxyvitamin D”, “calcitriol”, “measurement”, “LC-MS/MS”, “chromatography”, “immunoassay”, “derivatisation”, “immunoaffinity extraction”). Boolean operators were used to structure the search strategy. Synonyms within each concept (e.g., "calcitriol", "1,25-dihydroxyvitamin D") were combined using OR, and distinct concepts (e.g., analyte, measurement technique) were combined using AND. Filters applied included English language and full-text availability. Additional pertinent articles were also identified and incorporated into this review through manual screening of the bibliographies of the identified papers.

Inclusion Criteria

The inclusion criteria adopted were (i) peer-reviewed original research, reviews, systematic reviews, and meta-analyses; (ii) articles examining or reporting analytical techniques for measuring serum 1,25(OH)_2_D concentration in human samples, including immunoassays, LC-MS/MS, HPLC, and other chromatographic or immunoanalytical methods; (iii) articles describing analytical validation or performance, reference range establishment, or standardisation initiatives for 1,25(OH)_2_D.

Exclusion Criteria

Articles were excluded if they (i) did not report or evaluate analytical methods for measuring serum 1,25(OH)_2_D in human samples; (ii) focused exclusively on other vitamin D metabolites without reference to 1,25(OH)_2_D; (iii) were non-primary research outputs such as abstracts, case reports, letters, or commentaries; (iv) were not published in English language.

Data Extraction

Data extraction from each article focused on analytical techniques reported in each study, including the type of assay used, sample preparation steps, and assay performance metrics (sensitivity, specificity, precision, and limits of detection). Assay standardisation initiatives were also included in this review. In studies reporting a comparison of two or more assay performance metrics for serum 1,25(OH)_2_D, data were tabulated to facilitate comparison of analytical approaches.

Data Synthesis

This review does not involve a meta-analysis due to the significant heterogeneity noted across the included studies. Sources of heterogeneity included differences in study populations and marked methodological inconsistencies within analytical methods. Populations ranged from adults to children, healthy controls to patient cohorts, and from individual specimens to pools. Analytical methods had marked variations in sample preparation, extraction techniques, and calibration protocols employed. Even among studies employing the same analytical technique, discrepancies in these domains persisted. These methodological and population-level differences limited the feasibility of deriving pooled effect estimates. Findings were therefore synthesised narratively to report key analytical advances for quantifying serum 1,25(OH)_2_D, while highlighting the strengths and limitations of available analytical techniques and the persisting gaps in standardisation and clinical application. Potential limitations of this approach and sources of bias are discussed in the following section.

Risk of Bias

This review was conducted as a narrative synthesis rather than a systematic review, and therefore, formal systematic review protocols or quality appraisal frameworks were not incorporated. The narrative approach was adopted, given the presence of significant heterogeneity across the studies. Therefore, the presence of selection bias cannot be excluded. Strict inclusion and exclusion criteria were applied; however, inclusion was based on the authors' judgement of relevance instead of a formal scoring framework. Literature searches were conducted using Ovid MEDLINE and Embase, which provide extensive coverage of relevant publications. Nevertheless, relevant articles published exclusively in other databases may still have been unintentionally excluded.

The risk of publication bias is considered low but cannot be completely dismissed. Unlike many research fields where positive findings can predominate, many studies included in this review reported inconsistent correlation between immunoassays and LC-MS/MS. It still remains possible that studies reporting poor assay performance may have been less likely to be published in the literature, skewing the interpretations drawn. Furthermore, industry-funded studies may be more likely to favour testing of particular analytical platforms, influencing the pool of published evidence.

Finally, interpretive bias must also be acknowledged. Narrative synthesis of the included studies involves a degree of subjectivity in reporting findings. Efforts were made to provide a balanced overview of the studies included. However, the interpretation of analytical methods may reflect the authors’ judgment. The conclusions should therefore be viewed as an informed synthesis of current analytical practices in quantifying 1,25(OH)_2_D rather than definitive evaluations, highlighting the need for continued research and better laboratory standardisation.

Radioreceptor assay (RRA)

Assay Principle

RRAs are competitive binding assays that quantify hormones or metabolites by measuring their ability to bind to a specific receptor in the presence of a radioactively labelled analogue [29,30].

Advances and Limitations

RRAs were the earliest methods developed for measuring serum concentration of 1,25(OH)_2_D_3_, with the first method introduced by Brumbaugh et al. in 1974 [29]. In this assay, 1,25(OH)_2_D_3_ was extracted from plasma using organic solvents and subjected to four chromatographic separation columns to isolate 1,25(OH)_2_D_3_ [29,30]. A radioactive tritium-labelled analogue (^3^H-1,25(OH)_2_D_3_) was added to compete with the unlabelled 1,25(OH)_2_D_3_ for binding to the VDR, sourced from chicken intestines [29]. The VDR-1,25(OH)_2_D_3_ complexes were retained using a glass filter, and radioactivity was quantified against an isotope dilution standard curve to estimate the concentration of bound 1,25(OH)_2_D_3_. The measured radioactivity was directly related to the amount of bound 1,25(OH)_2_D_3_ [29].

This assay was able to detect both the D_2_ and D_3_ metabolites, but the VDR sourced from chicken demonstrated a lower binding efficiency for the D_2_ metabolite [31]. Whilst this assay demonstrated acceptable levels of accuracy (±10% from known standards), imprecision (below 12% coefficient of variation (CV)) and sensitivity (detecting concentrations as low as 24 pmol/L or 10 pg/mL) [18,29,30], the assay's complexity and need for a substantial volume of plasma (≥20 mL) made it challenging to implement for clinical testing [18].

A second assay was later introduced by Eisman et al. to propose modifications to the original RRA method and address some of its limitations; this modified method required only 5 mL of plasma volume and made the purification phase less complex [32]. The proposed modifications involved incorporating an HPLC step to isolate 1,25(OH)_2_D [32]. However, the purification process still involved numerous steps to isolate 1,25(OH)_2_D from the remainder of vitamin D metabolites, particularly 25(OH)D, which circulates in higher concentrations and can interfere with measurements by binding to the VDR [18,32].

Multiple modifications were made to improve the original assay. Firstly, a modified solid-phase extraction (SPE) method was implemented to purify 1,25(OH)_2_D instead of HPLC [33], using dual silica and C-18 cartridges that enable both reverse-phase and normal-phase purification [34]. Later refinements streamlined this further by using only a single C-18 cartridge [35]. Secondly, this method also replaced the VDR obtained from chicks with one obtained from calf thymus, initially identified by Reinhardt et al. [36]. This alternate VDR displayed improved stability and equal affinity for both D_2_ and D_3_ metabolites [37]. These improvements enhanced assay sensitivity and specificity, rendering the initial HPLC purification step unnecessary and forming the basis for a simpler two-step SPE-based assay [33,34,38].

This method helped address limitations observed in previous assays; it required only 1 mL of plasma, which meant it was suitable for paediatric samples, and demonstrated an acceptable level of sensitivity (<4.8 pmol/L or <2 pg/mL) [18]. The SPE procedure, however, was very time-consuming [33,34]. In the years following, multiple different analytical methods that used either electrochemical detection or gas chromatography with mass spectrometry were developed and achieved similar sensitivity levels, but they were largely impractical and difficult to implement routinely because of the cumbersome analytical steps and high cost [39,40].

Immunoassays

Radioimmunoassay (RIA)

Assay principle: RIAs are competitive binding assays that use radiolabelled tracers and specific antibodies to quantify low-concentration analytes in biological samples [41].

Advances and limitations: The first evidence for using RIA to quantify serum 1,25(OH)_2_D was published in 1978 [4,41]. Early methods involved using antibodies against 25(OH)D to detect 1,25(OH)_2_D [41]. These antibodies, however, showed significant cross-reactivity with other vitamin D metabolites, particularly 25(OH)D and 24,25(OH)_2_D. Thus, additional chromatographic separation techniques remained necessary to isolate 1,25(OH)_2_D before measurement [41].

Hollis et al. introduced an improved RIA [42], which was later commercially presented as DiaSorin RIA. Instead of using HPLC, a dual column SPE procedure using a C-18-OH cartridge linked to a silica cartridge was incorporated to isolate 1,25(OH)_2_D from the plasma [42]. Plasma samples (≥0.5 mL) were extracted using an organic solvent and passed through the two SPE columns. To quantify 1,25(OH)_2_D, a competitive RIA procedure was utilised in which rabbit antibodies bound to 1,25(OH)_2_D competed with 1,25(OH)_2_D tagged with a radioactive iodine tracer (I-125) [42]. The degree of binding was measured using gamma counting [42].

This updated RIA approach presented several advantages. Firstly, it bypassed the requirement for HPLC purification. Secondly, it replaced the tritiated tracer used previously in RRAs with a radiolabelled one, which is more practical and allows for gamma counting. Thirdly, it decreased assay time to only five hours. Finally, it markedly addressed cross-reactivity with other metabolites. This assay displayed recovery of 90-101% for 1,25(OH)_2_D_3_ and 67-71% for 1,25(OH)_2_D_2_ [4], with a lower detection limit of 6.2 pmol/L (2.4 pg/mL) [4].

Following the introduction of the DiaSorin RIA to the market, another RIA procedure was introduced by Immunodiagnostic Systems (IDS) Ltd. (Boldon, UK). Unlike in DiaSorin, the 1,25(OH)_2_D extraction stage was done by a solid-phase monoclonal antibody [43]. Plasma samples were first evaporated to eliminate moisture and subsequently incubated with 1,25(OH)_2_D antibodies overnight [43]. A competitive RIA procedure was used, where a tracer labelled with I-125 and derived from 1,25(OH)_2_D was utilised [43]. This IDS RIA technique demonstrated a good level of reactivity towards both 1,25(OH)_2_D_2_ and 1,25(OH)_2_D_3_, at 79% and 100% respectively, while also requiring minimal sample volume (0.5 mL) [43]. Furthermore, cross-reactivity with other metabolites, particularly 25(OH)D_3_ and 24,25(OH)_2_D, was negligible [43].

Despite variations in their procedures, DiaSorin and IDS RIA demonstrated satisfactory precision, achieving recoveries up to 100% (± 17%) [42,43]. These assays were able to measure concentrations as high as 480 pmol/L or 200 pg/mL [42,43], with comparable lower detection limits (DiaSorin: 6.0 pmol/L or 2.4 pg/mL, IDS: 5 pmol/L or 2 pg/mL) [42,43]. Due to their practicality, both methods achieved widespread adoption in laboratories [18]. DiaSorin and IDS RIA were the principal approaches for measuring 1,25(OH)_2_D before being gradually superseded by LC-MS/MS and automated immunoassays.

Enzyme-Linked Immunoassay (EIA)

Since the early 2000s, EIAs have been available to measure the concentration of 1,25(OH)_2_D. EIA necessitates either manual setup or use of an automated liquid handler on a specialised assay platform [18]. One of the analytical methods utilising EIA was proposed by IDS [44]. The IDS EIA assay utilised an enzyme-tagged form of the molecule rather than a radiolabelled one to compete with 1,25(OH)_2_D for binding with the antibodies [44]. This method was shown to have excellent correlation with the RRA method using calf thymus; IDS EIA even demonstrated better precision [44]. However, EIA platforms had several limitations, including moderate imprecision and reduced reactivity to the 1,25(OH)_2_D_2_ metabolite, which can affect accuracy in clinical contexts [44].

Chemiluminescent Immunoassay (CLIA)

As clinical interest in quantifying 1,25(OH)_2_D increased, advancements have shifted towards developing automated immunoassays with reduced turnaround times to handle the increasing demand of clinical routine testing. DiaSorin and IDS were the main commercial vendors that produced analytical methods to address these demands. Both methods have largely replaced their RIA predecessors and continue to be used for clinical testing today. Of the two, DiaSorin LIAISON® XL is fully automated [45]; IDS-iSYS requires a manual pre-analytical extraction step before analysis [46].

DiaSorin LIAISON® XL: The DiaSorin LIAISON® XL utilises a three-step sandwich CLIA [45]. Firstly, the ligand binding domain (LBD) of a recombinant VDR is used to bind to the 1,25(OH)_2_D molecule, forming a receptor-1,25(OH)_2_D complex [45]. Secondly, the formed complexes are detected using a monoclonal antibody; the antibody detects the LBD of the complexes in the sample, effectively identifying the presence of 1,25(OH)_2_D [45]. Finally, a chemiluminescent chemical reaction is triggered, and the intensity of the emitted light is detected by a highly sensitive photomultiplier [45]. The intensity of the generated light corresponds directly to the quantity of complexes present. This assay does not require a manual immunoextraction step as the VDR used is highly specific to 1,25(OH)_2_D. The lower limit of quantification (LLoQ) quoted for this DiaSorin immunoassay is 12 pmol/L (5 pg/mL) [46].

IDS-iSYS: By contrast, the first step of the IDS-iSYS assay involves a manual immunoextraction process, wherein the sample is incubated with magnetic particles coated with antibodies that specifically target 1,25(OH)_2_D [46]. The sample is then transferred to the IDS-iSYS Multi-Discipline Automated System, where it is incubated with sheep anti-1,25(OH)_2_D antibodies followed by an acridinium-labelled 1,25(OH)_2_D conjugate [46]. Thirdly, a chemiluminescent reaction is triggered, whereby the intensity of the generated light is inversely proportional to the concentration of 1,25(OH)_2_D, unlike in DiaSorin [46]. IDS-iSYS exhibits an LLoQ of 18 pmol/L (7.5 pg/mL) [46]. Compared to IDS RIA, IDS-iSYS was quoted to obtain measurements that are lower by an average of 20% [46].

LC-MS/MS

Background: Historically, the accessibility of LC-MS/MS was confined to certain well-equipped laboratories. However, due to significant advancements over recent years, it has become more accessible across various laboratory settings. While automated immunoassays have been commonly used to quantify the concentration of 1,25(OH)_2_D, they are still prone to limitations that can introduce measurement biases. The antibodies used by immunoassays are prone to cross-reacting with other vitamin D metabolites [18], and some may have variable affinities to both the D_2_ and D_3_ forms of 1,25(OH)_2_D; particularly, they have been observed to generally possess lower affinities for 1,25(OH)_2_D_2_ [18]. As a result, these interferences may result in positive (overestimation) or negative (underestimation) biases in the measurements.

Assay principle: LC-MS/MS has the capacity to differentiate and quantify individual vitamin D metabolites, including distinguishing between the D_2_ and D_3_ forms, by detecting their unique mass-to-charge (m/z) transitions during mass spectrometry analysis [47,48]. As a result, LC-MS/MS-based methods do not necessitate intricate purification processes, unlike immunoassays. Nevertheless, as previously mentioned, the inherent lipophilic nature of the 1,25(OH)_2_D molecule still gives rise to a considerable challenge in its quantification using LC-MS/MS due to its low ionisation efficiency [49].

Strategies to enhance detection by LC-MS/MS: Several analytical techniques have been developed to either directly improve the molecule’s ionisation efficiency or enhance the sensitivity of LC-MS/MS to 1,25(OH)_2_D. One approach involves adding certain additives, such as ammonium or lithium acetate, to facilitate the formation of more stable ion species during the ionisation stage of mass spectrometry, thereby enhancing detection sensitivity [50]. The sample is then inserted into a dual-column chromatography system, which further enhances separation efficiency and sensitivity to analytes [51]. Another strategy involves performing a manual immunoaffinity extraction procedure before subjecting the sample to LC-MS/MS analysis, which has also been demonstrated to enhance assay sensitivity [25]. A third widely used technique to enhance the ionisation efficiency of 1,25(OH)_2_D before mass spectrometry analysis is to derivatise the sample [25]. Derivatisation involves chemically modifying the 1,25(OH)_2_D molecule by adding polar groups to it through specific reagents, forming derivatives that have superior ionisation properties compared to the original molecule [52]. The more stable derivatives, in turn, allow for improved mass spectrometry detection and sensitivity [52].

LC-MS/MS With Two-Dimensional Chromatography

An early LC-MS/MS approach proposed to improve the ionisation of 1,25(OH)_2_D involved the use of ammonium acetate [50]. This method was tested on pig and rat serum and involved lengthy pre-analytical steps while also suffering from a lack of sensitivity [50]. To address these limitations, several analytical modifications to this method followed; most notably, the lengthy pre-analytical steps were significantly simplified with the introduction of online two-dimensional chromatography as demonstrated by Casetta et al. [51].

In this refined method, proteins were first removed from the sample through precipitation, followed by a two-step chromatographic process [51]. Lithium acetate was added during the mobile phase, leading to the formation of lithium adduct ions inside the mass spectrometer during electrospray ionisation (ESI) [51,53]. The mass spectrometer is set to monitor the precursor lithium adduct ions of 1,25(OH)_2_D and the specific product ions via multiple reaction monitoring (MRM) [51,54]. This method demonstrated improved sensitivity compared to the original assay; it had a LLoQ of 36 pmol/L (15 pg/mL) [18,51].

LC-MS/MS With Manual Immunoaffinity Extraction

An alternative strategy to improve LC-MS/MS sensitivity was proposed by Yuan et al., who used immunoaffinity extraction before analysis [25,55]. Unlike the two-dimensional chromatography method described by Casetta et al., this approach relied solely on immunoaffinity extraction without additional pre-analytical steps. In this method, serum samples were initially incubated for one hour in Immunotube® (Immundiagnostik AG, Bensheim, Germany) columns containing an antibody slurry to selectively bind to 1,25(OH)_2_D [55]. After washing and elution, the isolated compound was introduced into the LC-MS/MS system [55]. Inside the chromatography column, lithium acetate was again included in the mobile phase to facilitate the formation of lithium adduct ions during ESI [55]. Yuan et al.’s LC-MS/MS assay displayed excellent sensitivity in quantifying both 1,25(OH)_2_D_3_ and 1,25(OH)_2_D_2_, achieving a detection limit below 9.6 pmol/L (4 pg/mL) for both [18,55].

A key limitation of this method was the potential for inconsistencies resulting from the antibodies used. Because there were no other pre-analytical steps, the antibodies used during the extraction step were mainly responsible for the performance of the assay [55]. Different antibodies had variable performance; for instance, when mouse antibodies were used for immunoaffinity extraction, mass spectrometry results failed to quantify 1,25(OH)_2_D [55,56]. In contrast, Strathmann et al. demonstrated better selectivity and a reduction in chromatographic separation times when using a solid-phase antibody instead [56].

LC-MS/MS With Derivatisation

A key strategy to enhance LC-MS/MS sensitivity is through derivatisation, which involves chemically modifying 1,25(OH)_2_D to improve its ionisation efficiency and detection accuracy during LC-MS/MS analysis [52]. Several derivatising agents have been used for 1,25(OH)_2_D. PTAD (4-phenyl-1,2,4-triazoline-3,5-dione) was used by Aronov et al. to derivatise 1,25(OH)_2_D_3_ and 1,25(OH)_2_D_2_, achieving a 100-fold increase in sensitivity [57]. Their method involved extraction and purification steps prior to derivatisation, but the analysis process took approximately 11 minutes to complete [57]. The reported detection limit for both 1,25(OH)_2_D_2_ and D₃ remained relatively high, at 60 pmol/L (25 pg/mL) for this method [18,57]. Despite the extraction techniques employed, there were concerns regarding the presence of lipids in the samples even after the extraction process, which could have interfered with the analysis by causing matrix suppression during mass spectrometry [57].

To address these limitations, Duan et al. introduced a modified version of the assay combining PTAD derivatisation with a nano-LC-MS/MS setup [58]. Their method significantly enhanced sensitivity, reporting an LLoQ of 12 pmol/L (5 pg/mL) [58]. However, each sample required 27 minutes from injection to completion, limiting its suitability for high-volume testing. Hedman et al. attempted to test another novel derivatising agent, Amplifex Diene (AB SCIEX), before LC-MS/MS analysis [59]. They compared measurements obtained using Amplifex Diene with those obtained using PTAD on 20 different patient samples [59]. Amplifex offered superior sensitivity (LLoQ: 4.8 pmol/L or 2 pg/mL) and a 10-fold increase in signal-to-noise ratio [59]. Similarly, Wan et al. tested another derivatising agent (2-nitrosopyridine) and evaluated its performance relative to PTAD [60]. 2-nitrosopyridine was better than PTAD in improving the ionisation efficiency of 1,25(OH)_2_D as it yielded a 10-fold increase in sensitivity [60].

Interference by Circulating Moieties

**Figure 4:**
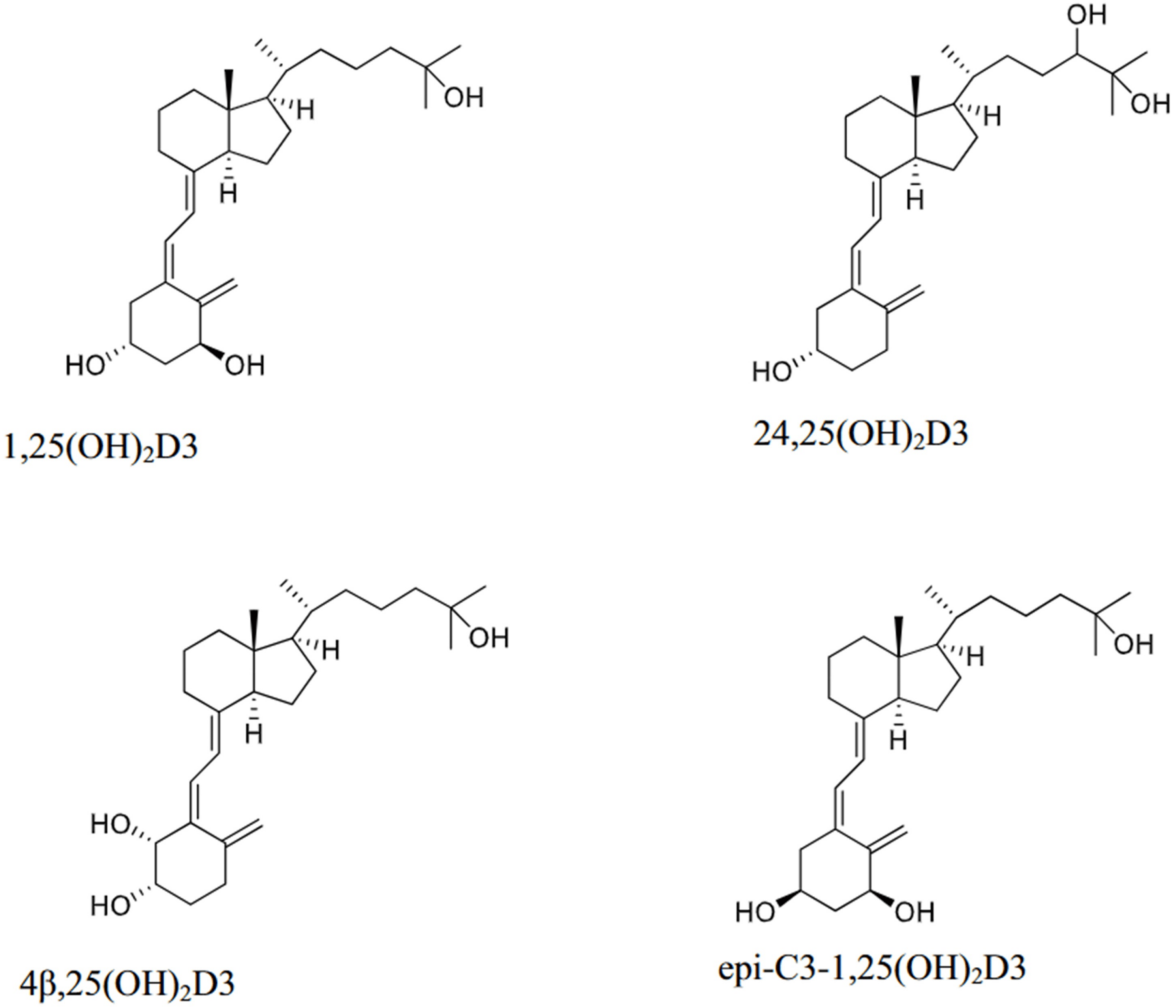
Structures of 1,25-dihydroxyvitamin D₃ and three interfering metabolites commonly measured during LC-MS/MS analysis: 24,25(OH)₂D₃, 4β,25(OH)₂D₃, and epi-C3-1,25(OH)₂D₃. Reproduced from Reference [18]. ^©^Taylor & Francis Ltd. Reprinted with permission of the publisher (Taylor & Francis Ltd., http://www.tandfonline.com). License No. 6050390763864.

In addition to the low ionisation energy of 1,25(OH)_2_D, an additional analytical challenge arises from interference by circulating vitamin D isomers. Without adequate chromatographic separation or purification, these compounds can lead to inaccurate quantification [18,61,62]. The most commonly cited interfering isomers are shown in Figure 4.

Conventional liquid chromatography prior to mass spectrometry can separate 1,25(OH)_2_D_3_ from 25(OH)D_3_ and 24,25(OH)_2_D_3_. However, other metabolites like 3-epi-1,25(OH)_2_D_3_ and 4β,25(OH)_2_D_3_ require additional analytical strategies, such as chromatographic separation or immunoaffinity steps [18]. 3-epi-1,25(OH)_2_D_3_ arises through an alternative metabolic pathway of 1,25(OH)_2_D_3_ and exhibits similar, though not identical, biological activity [61,63]. In contrast, 4β,25(OH)_2_D_3_ is produced by the liver microsomes when the parent metabolite (25(OH)D_3_) is hydroxylated [64]. Both isomers are present in circulation in the picomolar range, similar to 1,25(OH)_2_D_3_, and can interfere with measurements obtained by LC-MS/MS if not accounted for [63,64].

One strategy to mitigate interference from structurally similar isomers, as shown by Wang et al., is to extend the chromatography duration to allow for the separation of these isomers [64]. Another strategy is to perform an immunopurification step prior to LC-MS/MS analysis, as discussed earlier [56]. Whilst immunopurification can effectively isolate 1,25(OH)_2_D from most isomers, it is less efficient in removing 24,25(OH)_2_D [56]. Despite this apparent limitation, fragmentation of 24,25(OH)_2_D in mass spectrometry analysis yields a different product ion and is unlikely to interfere with the detection of 1,25(OH)_2_D [56].

Comparing LC-MS/MS to automated immunoassays

LC-MS/MS is widely regarded as the “gold-standard” method for measuring 1,25(OH)_2_D as it offers higher levels of accuracy and specificity compared to immunoassays [4,18,25]. However, automated CLIAs have been widely used by many laboratories, particularly DiaSorin LIAISON® XL and IDS iSYS, as they offer convenience and ease of use in high-volume testing [25]. Several studies have attempted to compare the performance of these automated immunoassays against LC-MS/MS by testing the same set of samples (Table 1). Zittermann et al. measured 1,25(OH)_2_D concentrations in 129 adult serum samples obtained from cardiac patients using both DiaSorin immunoassay and an LC-MS/MS method involving Immunotubes® for pre-analysis extraction [65]. Measurements from both methods correlated poorly, as indicated by a correlation coefficient (r) value of 0.534. The mean 1,25(OH)_2_D concentration obtained from immunoassay was 9.5 pmol/L (3.8 pg/mL) higher than that of LC-MS/MS [65].

**Table 1:** Comparison between the performance of immunoassays with LC-MS/MS in the literature. LC-MS/MS: liquid chromatography-tandem mass spectrometry For consistency, r values are shown throughout the table. Original studies that reported r^2^ were converted to r.

Authors	Population	Comparison	Mean Bias (95% Limits of Agreement)	Regression Analysis
Zittermann et al. [65] (2016)	129 adult samples	DiaSorin LIAISON^®^ XL and LC-MS/MS	-12.8% (-96% to 70.3%)	y = 1.27x - 1.99 (r = 0.534)
Spanaus and von Eckardstein [46] (2017)	142 adult samples	DiaSorin LIAISON^®^ XL and LC-MS/MS	2.3% (-29.2% to 33.7%)	y = 1.03x - 0.73 (r = 0.97)
		IDS-iSYS and LC-MS/MS	7.0% (-69.8% to 83.9%)	y = 1.34x - 9.18 (r = 0.85)
Valcour et al. [66] (2016)	78 adult samples	DiaSorin LIAISON^®^ XL and LC-MS/MS (method 1)	2.4% (-22.5% to 27.3%)	y = 0.98x + 1.93 (r = 0.92)
		DiaSorin LIAISON^®^ XL and LC-MS/MS (method 2)	15.5% (-8.1% to 39.2%)	y = 1.07x + 3.77 (r = 0.94)
Higgins et al. [67] (2018)	7 paediatric samples	DiaSorin LIAISON^®^ XL and LC-MS/MS	8.8% (range: 0% to 26.5%)	y = 1.212x - 30.7 (r = 0.98)
Tang et al. [68] (2024)	80 adult and 422 paediatric samples	DiaSorin LIAISON^®^ XL and LC-MS/MS	Adult samples: -1.6 (±14.3, -29.6 to 26.5) pmol/L; Paediatric samples: -9.8 (±23.4, -55.7 to 35.9) pmol/L	Adult samples: y = 0.86x + 11.48 (r =0.97); Paediatric samples: y = 0.81x +15.44 (r =0.81)

Conversely, Spanaus and von Eckardstein reported excellent correlation between DiaSorin immunoassay and LC-MS/MS (r = 0.97) when testing 142 adult serum samples [46]. In contrast, the IDS-iSYS immunoassay showed weaker correlation with LC-MS/MS (r = 0.85) [46]. The mean bias between DiaSorin and LC-MS/MS measurements was at only 2.3% (95% limits of agreement (LoA) -29.2% to 33.7%), whereas it was much larger between IDS-iSYS and LC-MS/MS measurements at 7.0% (95% LoA -69.8% to 83.9%); IDS-iSYS overestimated measurements at higher 1,25(OH)_2_D concentrations [46]. Additionally, DiaSorin demonstrated superior precision (CV ≤5.2%) compared to IDS-iSYS (CV of up to 20.1%) [46].

Valcour et al. compared the DiaSorin immunoassay to two different LC-MS/MS assays that both utilised an immunoextraction step [66]. The immunoassay exhibited good correlation (r = 0.92) with one of the LC-MS/MS methods, yielding the regression equation $y = 0.98x + 1.93$ (95% confidence interval (CI) for the slope 0.90-1.06; 95% CI for the intercept -1.81 to 5.67) [66]. The mean bias between immunoassay and LC-MS/MS was 2.4% (95% CI -0.5% to 5.2%; 95% LoA -22.5% to 27.3%) [66]. Compared to the other LC-MS/MS method, immunoassay displayed slightly better correlation (r = 0.94) with the equation $y = 1.07x + 3.77$ (95% CI for the slope 1.00-1.15; 95% CI for the intercept 0.41-7.13) [66]. The mean bias, however, was substantially higher at 15.5% (95% CI 12.8%-18.3%; 95% LoA -8.1% to 39.2%) [66].

Higgins et al. analysed 1,25(OH)_2_D levels in seven pooled serum samples from neonates and infants using both LC-MS/MS and the DiaSorin LIAISON® XL immunoassay [67]. Both methods displayed a strong linear relationship, yielding the Deming regression equation $y = 1.212x - 30.7$ (r = 0.98) [67]. However, a concentration-dependent positive bias was observed, with the immunoassay overestimating measurements at higher serum 1,25(OH)_2_D concentrations [67]. The greatest discrepancy occurred in the sample with the highest concentration pool, where DiaSorin reported 640.5 pmol/L compared to 506.5 pmol/L by LC-MS/MS, constituting a 26.5% bias [67]. The observed difference was initially attributed to interference from 3-epi-1,25(OH)_2_D; however, it was undetectable in all samples, suggesting interference by another unidentified moiety [67].

Tang et al. used both LC-MS/MS and the DiaSorin LIAISON® XL to assess serum 1,25(OH)_2_D concentrations in 80 adult and 422 paediatric samples [68]. While both methods correlated well for the adult samples (r =0.97), a weaker correlation was noted in the paediatric cohort (r=0.81); the immunoassay showed a mean negative bias of 9.8 pmol/L (±23.4), with 95% LoA ranging from -55.7 to +35.9 pmol/L [68]. Surprisingly, unlike the findings of Higgins et al. [67], the variability observed in the paediatric samples was not dependent on either the serum 1,25(OH)_2_D concentration or participant age [68].

In summary, current evidence demonstrates variable agreement between automated immunoassays and LC-MS/MS. DiaSorin LIAISON® XL generally shows stronger correlation with LC-MS/MS, though discrepancies have been shown at high 1,25(OH)_2_D concentrations and in paediatric samples [46,67,68]. IDS-iSYS tends to exhibit greater bias and weaker correlation to LC-MS/MS in comparison [46]. These findings highlight the inherent limitations of cross-reactivity in antibody-based immunoassays [4,18], reinforcing the role of LC-MS/MS as a reference method. The notable variability in serum 1,25(OH)_2_D measurements across different assays reflects broader challenges in standardisation, discussed in the following section.

Standardisation efforts for 1,25(OH)_2_D

The variability of measurements obtained from different assays highlights the ongoing challenges in measuring 1,25(OH)_2_D. Variations can be observed not only between different assays, but also between different laboratories using the same assay [26]. Due to the absence of NIST standards and an RMP for 1,25(OH)_2_D, comparison of measurements by different assays across different laboratories remains challenging [4,26].

In the absence of an international standardisation scheme for 1,25(OH)_2_D, several initiatives have emerged to mitigate inter-laboratory variability. First, several groups working with the CDC have developed isotope-dilution LC-MS/MS methods with immunoaffinity enrichment and stable isotope-labelled internal standards for 1,25(OH)_2_D [56]. These methods were designed to improve analytical specificity and accuracy, mitigating the limitation of cross-reactivity with immunoassays [46,58]. Second, DEQAS contributes to the standardisation of 1,25(OH)_2_D by providing external quality control materials to participating laboratories [27]. Laboratories analyse these samples using their own assays and submit the results to DEQAS, which compares them against the method mean or the all-laboratory trimmed mean (ALTM) rather than to an established RMP [26]. Immunoassays (particularly IDS and DiaSorin) constitute the majority of the submissions; thus, concerns regarding immunoassays skewing the ALTM and masking method-specific bias exist [25,27]. Significant variability in the mean 1,25(OH)_2_D concentrations reported by different methods on the same samples also exists, and the CVs for different laboratories using the same method are highly variable [26,27]. Furthermore, the annual DEQAS proficiency criteria for 1,25(OH)_2_D are quite broad, limiting comparability; to pass, an assay is required to produce 80% of its annual measurements (on DEQAS samples) within ±30% of the ALTM [27]. Despite these limitations, DEQAS participation offers laboratories an opportunity to monitor performance relative to peers and identify systematic biases.

These initiatives highlight recognition of the problem and are important steps towards achieving standardisation for 1,25(OH)_2_D. However, they remain fragmented and are insufficient compared to the structural framework in place for 25(OH)D. The lack of reference materials and endorsed RMPs continues to hinder comparability across laboratories to ensure clinically reliable and reproducible measurements for 1,25(OH)_2_D.

Clinical implications

In clinical practice, immunoassays, particularly the fully automated DiaSorin LIAISON® XL, remain the mainstay for quantifying 1,25(OH)_2_D. Current reference ranges for 1,25(OH)_2_D are method-specific and largely determined from immunoassay-generated data [65,69]. This widespread reliance on immunoassay-derived testing for 1,25(OH)_2_D persists despite well-documented limitations with cross-reactivity and lack of specificity [18], which contribute to measurement variability. Combined with a lack of laboratory standardisation, such variability introduces a significant risk of misinterpretation, putting diagnostic accuracy at risk. These concerns are particularly pronounced in paediatric settings, where physiologically elevated levels of 1,25(OH)_2_D exist in early life [65]. In this context, measurements obtained via immunoassays have been shown to significantly overestimate true concentrations compared to LC-MS/MS, likely to result from interference by structurally similar metabolites [65]. The need for standardised measurement protocols and LC-MS/MS-based reference intervals for 1,25(OH)_2_D remains essential to support reliable clinical interpretation.

## Conclusions

1,25(OH)_2_D has emerged as a key biomarker not only in maintaining several physiological processes, but also as a diagnostic tool in disorders of bone metabolism, chronic kidney disease, and certain endocrine and inflammatory conditions. The field of quantifying 1,25(OH)_2_D continues to advance, with several assays available on the market today. Given the number of assays available, variations have been observed across different assays and laboratories. While most laboratories prefer automated immunoassays due to their convenience and capacity to meet the continuous demand, they may suffer from limitations related to specificity, cross-reactivity, and inter-assay variability. In contrast, LC-MS/MS remains the gold standard due to its superior specificity and ability to distinguish closely related vitamin D metabolites, but its wider implementation is limited by cost and throughput constraints.

Future work should prioritise the generation of robust reference intervals using LC-MS/MS-based data to support more accurate clinical interpretation of 1,25(OH)_2_D concentrations. The persisting challenge of methodological variations and the absence of a standardisation scheme and reference materials highlight the necessity for continued research in the field.
